# Draft Genome Sequence of *Coniochaeta ligniaria* NRRL 30616, a Lignocellulolytic Fungus for Bioabatement of Inhibitors in Plant Biomass Hydrolysates

**DOI:** 10.1128/genomeA.01476-16

**Published:** 2017-01-26

**Authors:** Diego Javier Jiménez, Ronald E. Hector, Robert Riley, Anna Lipzen, Rita C. Kuo, Mojgan Amirebrahimi, Kerrie W. Barry, Igor V. Grigoriev, Jan Dirk van Elsas, Nancy N. Nichols

**Affiliations:** a Cluster of Microbial Ecology, Groningen Institute for Evolutionary Life Sciences, University of Groningen, Groningen, The Netherlands; b Bioenergy Research Unit, National Center for Agricultural Utilization Research, USDA-ARS, Peoria, Illinois, USA; c U.S. Department of Energy Joint Genome Institute, Walnut Creek, California, USA

## Abstract

Here, we report the first draft genome sequence (42.38 Mb containing 13,657 genes) of *Coniochaeta ligniaria* NRRL 30616, an ascomycete with biotechnological relevance in the bioenergy field given its high potential for bioabatement of toxic furanic compounds in plant biomass hydrolysates and its capacity to degrade lignocellulosic material.

## GENOME ANNOUNCEMENT

*Coniochaeta ligniaria* is an ascomycete (order Coniochaetales), inhabiting decaying wood, leaf litter, and soil (1). *C. ligniaria* NRRL 30616 was isolated from furfural-contaminated soil based on its ability to metabolize furan-aldehyde mixtures (2). This strain has the potential to remove a variety of inhibitory compounds (e.g., 5-hydroxymethylfurfural) from plant biomass (e.g., wheat straw, switchgrass, corn stover, alfalfa stems, and rice hulls) dilute-acid hydrolysates, facilitating subsequent microbial fermentation of sugars (3–6). Moreover, *C. ligniaria*–like isolates have also been recovered from torrefied grass (7) as well as from various soil-derived lignocellulolytic microbial consortia (8, 9). Previous studies revealed that *C. ligniaria* contains key enzymatic machinery that efficiently works in lignocellulose deconstruction (10, 11). However, direct confirmation of the genomic potential has until now been missing.

To support information about the metabolism of furanic compounds and degradation of lignocellulosic biomass, we report here the draft genome sequence of *C. ligniaria* NRRL 30616. The strain was cultivated in yeast extract-peptone-dextrose (YPD) broth containing 50 μg/ml kanamycin. Total genomic DNA extraction was performed using the OmniPrep kit for fungi (G-Biosciences, St. Louis, MO). The genome was sequenced using the Illumina HiSeq 2000 platform at the Joint Genome Institute (JGI). The obtained quality reads were assembled with AllPathsLG version R47710 (12). The size of the assembled genome is 42.38 Mb (94.4× coverage), comprising 135 scaffolds (118 with more than 2 kb) and 230 contigs. The three largest scaffolds had 4.64, 4.17, and 3.94 Mb. Fungal genome annotation was performed using the JGI pipeline and is available via the JGI-MycoCosm platform (13). A total of 13,657 genes were predicted. Analysis of the genes with the CAZy database (14) identified 304 glycoside hydrolases, 100 glycosyl transferases, seven polysaccharide lyases, 45 carbohydrate esterases, 92 carbohydrate-binding modules, and 23 lytic polysaccharide monooxygenases (LPMOs) (AA9 and AA11 families), a new type of copper-dependent metalloenzymes that catalyze the oxidative cleavage of (1-4)-linked glycosidic bonds of plant polysaccharides and chitin (15). Regarding genes that could be involved in furanic compound metabolism (16), the *C. ligniaria* NRRL 30616 genome was found to contain 1,070 oxidoreductases, 926 dehydrogenases, and 227 decarboxylases. Based on gene ontology analysis, 23 genes are involved in the response to oxidative stress (GO:0006979).

The genomic information in this report will provide a better understanding of the genetic mechanism involved in the bioabatement of inhibitory by-products on plant biomass hydrolysates. In addition, the plethora of enzymes involved in lignocellulose degradation could be a relevant source for the production of new proteins useful in efficient saccharification of plant biomass. The availability of a genetic system for modification of *C. ligniaria* NRRL 30616 could enable engineering of the strain for conversion of biomass sugars to any number of value-added products.

### Accession number(s).

This whole-genome shotgun project has been deposited at DDBJ/ENA/GenBank under the accession no. MNPN00000000. The version described in this paper is version MNPN01000000.
